# Characterization of the blood microbiota in children with Celiac disease

**DOI:** 10.1016/j.crmicr.2021.100069

**Published:** 2021-08-30

**Authors:** Isha Mehrotra, Gloria Serena, Murat Cetinbas, Victoria Kenyon, Victoria M. Martin, Stephanie G. Harshman, Ali R. Zomorrodi, Ruslan I. Sadreyev, Alessio Fasano, Maureen M. Leonard

**Affiliations:** a Center for Celiac Research and Treatment, MassGeneral Hospital for Children, Yawkey Center for Outpatient Care, Suite 6B, 32 Fruit Street, Boston, MA 02114, USA; b Mucosal Immunology and Biology Research Center, MassGeneral Hospital for Children, Jackson, 55 Fruit Street, Boston, MA 02114, USA; c Celiac Research Program, Harvard Medical School, Boston, MA, USA; d Harvard Medical School, 25 Shattuck St, Boston, MA 02115, USA; e Department of Molecular Biology, Massachusetts General Hospital, Simches Research Center, 185 Cambridge Street, CPZN7250 Boston, MA 02114, USA; f Division of Pediatric Gastroenterology and Nutrition, MassGeneral Hospital for Children, Yawkey Center for Outpatient Care, Yawkey 6B-6800 Suite, 55 Fruit Street Boston, MA 02114, USA; g Food Allergy Center, Massachusetts General Hospital, MGH Professional Office Building, Suite 530, 275 Cambridge Street Boston, MA 02114, USA; h Eating Disorders Clinical and Research Program, Massachusetts General Hospital: West End Apartments 2 Longfellow Place, Suite 200 Boston, MA 02114, USA; i Neuroendocrine Unit, Massachusetts General Hospital, 100 Blossom St, Boston, MA 02114, USA

**Keywords:** Hematic, Coeliac, Microbiome, Pediatric, Bacteria, CD, Celiac disease, tTG-IgA, Tissue TransGlutaminase ImmunoGlobulin A, OTUs, operational taxonomic units

## Abstract

Celiac Disease (CD) is an autoimmune disorder triggered by gluten ingestion that can develop in genetically predisposed individuals. Alterations in the gut microbiota have been suggested to contribute to development of autoimmune conditions including CD. Recent work suggests the existence of a blood microbiota. Evidence that alterations in the blood microbiota potentially influence the development of chronic immune based diseases is increasing. However, there is no published literature regarding the blood microbiota in children, including those with CD. This study aimed to characterize the diversity and taxonomic composition of the blood microbiota of children with CD compared to controls. Whole blood samples were collected from children with active CD, CD in remission, and control subjects and 16S rRNA sequencing was utilized to analyze the blood microbiota. We found 16s rRNA present throughout all pediatric blood samples, providing evidence for the presence of a pediatric blood microbiota. We found significant differences in beta diversity and in abundance of certain taxa (Campylobacterales order, Odoribacteraceae and Helicobacteraceae families, *Odoribacter* genus and species, and *Bacteroides acidifaciens* species) between subjects with active CD and controls. These taxa have been previously reported to be associated with immune response and gut-inflammatory diseases. We did not find significant differences between subjects with active and remission CD or between remission CD and controls. Conclusions: We provide evidence for a pediatric blood microbiota and identified higher beta diversity and alterations in the composition of blood microbiota in subjects with active CD compared to controls.

## Highlights

• Bacteroidetes is most abundant phylum in pediatric blood.
• Beta diversity is increased in blood of children with active Celiac.
• Abundance of taxa are altered in blood of children with active Celiac.

## 1 Introduction

Celiac Disease (CD) is an autoimmune enteropathy triggered by gluten ingestion in genetically predisposed individuals. Genetic predisposition is necessary but insufficient for disease development (Salazar et al., 2017). The rapidly rising CD prevalence (Gatti et al., 2020) suggests environmental factors contribute to CD onset. Identification of these factors could help explain what triggers CD.

Due to the interaction of the gut microbiota with the immune system, alterations in the gut microbiota have been implicated in many chronic immune-based diseases (Kho and Lal, 2018) including CD (Chander et al., 2018; Leonard et al., 2020). Studies have identified an altered gut microbiota in children with CD compared to controls in stool (Di Cagno et al., 2011; Collado et al., 2008) and duodenal (Di Cagno et al., 2011; Collado et al., 2008) samples.

Recent literature has suggested the presence of a blood microbiota (Nikkari et al., 2001; Gosiewski et al., 2017; Paisse et al., 2016; Whittle et al., 2018), which has been hypothesized to originate from the gut microbiota (Paisse et al., 2016; Castillo et al., 2019). Blood was canonically considered sterile, but several studies, many utilizing 16S rRNA sequencing, have confirmed that blood from healthy individuals contains bacterial DNA (Nikkari et al., 2001; Gosiewski et al., 2017; Paisse et al., 2016), and identified distinct bacteria phyla (Paisse et al., 2016).

Alterations in the blood microbiota have been linked (Castillo et al., 2019) to chronic diseases including diabetes, cardiovascular disease, cirrhosis, and CD (Serena et al., 2019). Our lab previously utilized 16S rRNA sequencing to characterize taxonomic composition of the blood microbiota in adult patients with active CD, CD in remission, and controls (Serena et al., 2019) and identified alterations in blood of patients with active or remission CD compared to control adults.

While literature describing the adult blood microbiota is rapidly growing, to our knowledge, there is no published literature describing the pediatric blood microbiota. This study aimed to characterize diversity and taxonomic composition of the blood microbiota in children with active CD, CD in remission, and controls. In adults, the blood microbiome of patients with CD, controls, and those in remission were different in composition and diversity. Given the differences in adults, we anticipated that children may also have a blood microbiota and that there would be differences between children with CD, CD in remission, and controls. Specifically, we expect to identify differences in alpha and/or beta diversity, and in composition (abundance of certain taxa being different between the groups).”

While the blood microbiota field is controversial due to the longstanding belief that blood is sterile, investigation in CD is particularly interesting due to the hypothesis that the blood microbiota may be derived from the intestinal microbiota (Paisse et al., 2016; Castillo et al., 2019). Increased intestinal permeability is crucial in CD pathogenesis (Fasano, 2012) and subsequently, bacteria may be able to migrate and reach the bloodstream through this mechanism. Thus, it is possible that patients with CD, who have an altered gut microbiota, also have an altered blood microbiota (Castillo et al., 2019).

If indeed there are alterations in the blood microbiome associated with active CD, the blood microbiome could be utilized as a biomarker for CD. Further, it is plausible that an altered blood microbiome, due to blood circulation, an altered blood microbiota may contribute to some of the extraintestinal manifestations of CD

## 2 Materials and methods

### 2.1 Sample collection

Whole blood was collected from pediatric patients during clinically indicated endoscopies at MassGeneral Hospital for Children. We conducted a case control study, including sex-matched samples from subjects two to 16 years of age. Parents of children whose samples were collected provided written informed consent for the study and children age 7 and older provided assent, in concordance with guidelines from the Partners Human Research Committee Institutional Review Board. Samples were selected from 20 control subjects, 20 subjects with active CD, and 20 subjects with CD in remission on a Gluten Free Diet (GFD (Table 1). Patients with autoimmune or active gastrointestinal diseases other than CD such as type 1 diabetes, inflammatory bowel disease, and eosinophilic esophagitis and patients taking oral immunosuppressant medications were excluded from the study. We followed the same blood collection and measurement protocol previously published (Serena et al., 2019). To avoid contamination during the blood drawing, blood processing, and sequencing steps, sterile technique, sterile reagents, and sterile equipment was utilized.

**Table 1:** Study subjects’ metadata.

	Active CD (*n* = 20)	Remission CD (*n* = 20)	Control (*n* = 20)
Gender (%)			
Female	13 (65.0)	13 (65.0)	11 (55.0)
Age (%)			
	8.5 +/- 4.5 (2–16 years)	10.3 +/- 3.7 (5–16 years)	9.2 +/- 4.1 (2–16 years)
Marsh Score (%)			
0	0 (0.0)	12 (60.0)	
1	0 (0.0)	4 (20.0)	
2	0 (0.0)	4 (20.0)	
3A	2 (10.0)	0 (0.0)	
3B	13 (65.0)	0 (0.0)	
3C	5 (25.0)	0 (0.0)	
tTG IgA*			
	1019.9 (14.9–4965.5)	6.96 (1.9–21.8)	3.6 (1.9–13.9)
Length on GFD			
	.	2.1 (1–4.6 years)	.

*Reference range (0-20)

CD = Celiac Disease. *M* = Males. *F* = Female. GFD = Gluten Free Diet.

tTG IgA = Tissue TransGlutaminase ImmunoGlobulin A= IgA

*tTG IgA Reference range (0–20).

Pathology reports were reviewed from subjects during clinically indicated endoscopy procedures, and serum samples, collected for research purposes at the time of the procedure, were measured in our lab for Tissue Transglutaminase Immunoglobulin A (tTG-IgA) levels using QUANTA Lite Rh-tTG IgA ELISA (INOVA Diagnostics, San Diego, CA, USA) on a BioFlash machine. Serology and small intestinal histology were reviewed to characterize subjects for the study. Specifically, subjects with Marsh III pathology and tTG-IgA serology levels greater than 20 were diagnosed with CD and categorized as having active CD. Subjects previously diagnosed with CD who had been on a GFD for a minimum of one year, whose biopsies were determined to be Marsh 0, I, or II, indicating recovered mucosa, and whose tTG-IgA levels were less than or equal to 22 (negative cut-off value of 20), were categorized as in remission for CD. Subjects considered controls underwent clinically indicated endoscopies *for gastrointestinal symptoms such as abdominal pain or reflux and were found to have normal laboratory values, including* tTG-IgA levels below 14 (negative cut-off value or 20) *and histologically normal biopsies*.

### 2.2 DNA Extraction and amplification of hypervariable 16S gene region

The QIAamp DNA Blood Mini kit (Qiagen, Hilden, Germany) was used to extract total DNA from blood samples, following the manufacturer instructions. V4 hypervariable region of 16S rRNA gene was amplified by PCR. 806 reverse barcoded primers and 515 unique forward primers were used as previously described (Caporaso et al PNAS 2011). Gel electrophoresis confirmed amplification of the correct 16S rRNA gene region. The QIAquick PCR purification kit (Qiagen, Hilden, Germany) was used to purify PCR products following the manufacturer instructions. DNA concentration was measured by a Picogreen assay. A sample of DNAse/RNAse free water was processed and sequenced along the with the blood samples as negative control.

### 2.3 Illumina sequencing and computational analysis

Samples were sequenced at Massachusetts General Hospital Next-Generation Sequencing Core facility on the Illumina MiSeq instrument using MiSeq v2 500-cycle sequencing kit, resulting in approximately 25 million paired-end 250 base pair reads total (75,000 to 125,000 reads per sample) covering amplicon regions. The resulting fastq files were processed with QIIME software package (v. 2018.2.0) (Caporaso et al., 2010). The sequences with low quality score (on average less than 25) were truncated to 240bp and spurious reads were filtered using *deblur* algorithm with default settings (Amir et al., 2017). The remaining high quality sequences were aligned with *mafft* plugin (Katoh and Standley, 2013). Next, the aligned sequences were masked to remove highly variable positions and a phylogenetic tree is generated from the masked alignment by FastTree plugin (Price et al., 2010). Alpha and beta diversity metrics (Evenness-Group, Faith-Phylogenetic Diversity, Observed Operational Taxonomic Units (OTUs), Shannon-Group, Bray-Curtis, Jaccard, Unweighted and Weighted UniFrac) and Principal Component Analysis plots (based on Jaccard distance) were generated by default QIIME2 plugins (Caporaso et al., 2010; Lozupone and Knight, 2005; Lozupone et al., 2007; Chang et al., 2011; Chen et al., 2012; Vázquez-Baeza et al., 2013, 2017). To assign taxonomies to our sequences we have used QIIME2’s *feature-classifier* plugin and pre-trained Naïve Bayes classifier, which has been trained on the Greengenes 13_8 99% OTUs database. Differential abundance analysis of OTUs were performed by Linear Model as implemented in R package Maaslin2 (Mallick et al., 2021). Kruskal-Wallis test was used to assess statistical significance between alpha diversity group comparisons whereas Pairwise PERMANOVA test was used assess the statistical significance between beta diversity group comparisons (Anderson et al., 2011). Benjamini-Hochberg False Discovery Rate was employed for multiple testing corrections (Benjamini and Hochberg, 1995). The False Discovery Rate threshold level was set at 0.2.

## 3 Results

### 3.1 Alpha and beta diversity analysis

**Fig. 1:**
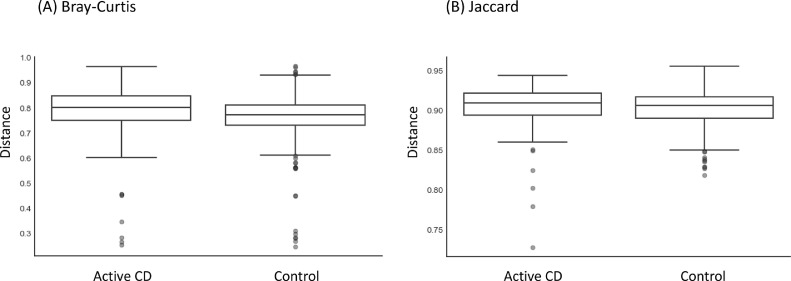
Beta diversity indices that differ between active CD and control subjects. Comparison of (A) Bray Curtis and (B) Jaccard Beta diversity indices between active CD subjects and control subjects. Boxplots indicate distances to active CD. Pairwise Permutational ANOVA tests were used to identify statistically significant differences between active CD and controls (Bray Curtis Index, *q* = .036; Jaccard index, *q* = .037).

We evaluated differences in alpha and beta diversity among the three groups: active CD, CD in remission, and controls. The analysis of alpha diversity measured using different metrics namely Evenness-Group index, Faith-Phylogenetic Diversity metric, Observed-Operational Taxonomic Units (OTUs) metric, and Shannon group, did not show any significant differences among groups. Comparisons between active CD and controls, however, identified significant differences in beta diversity as measured by Bray-Curtis (Fig. 1A, *q* = .036) and Jaccard (Fig. 1B, *q* = .037) indices, with the active CD group having higher beta diversity than controls (Fig. 1). Principal Component Analysis plots based on beta diversity metrics did not reveal any apparent clustering among the three groups (data not shown).

### 3.2 Microbiota composition analysis

**Fig. 2:**
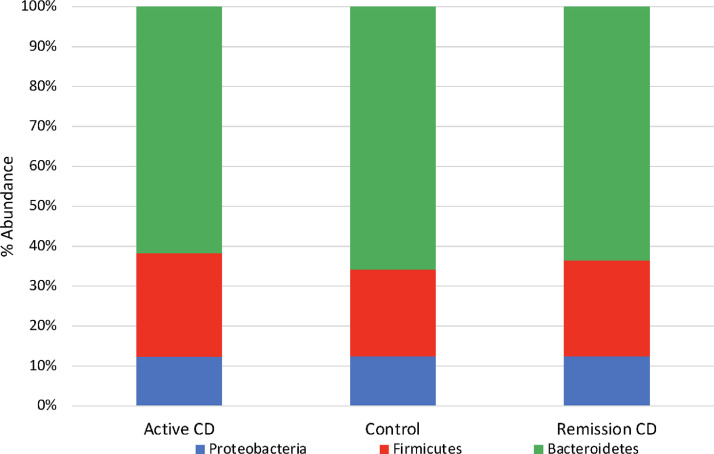
Most abundant phyla in active CD, remission CD, and control subjects. The top three most abundant phyla merged for the three groups are shown. Each bar shows the relative abundance of these three phyla among the samples in that group.

**Fig. 3:**
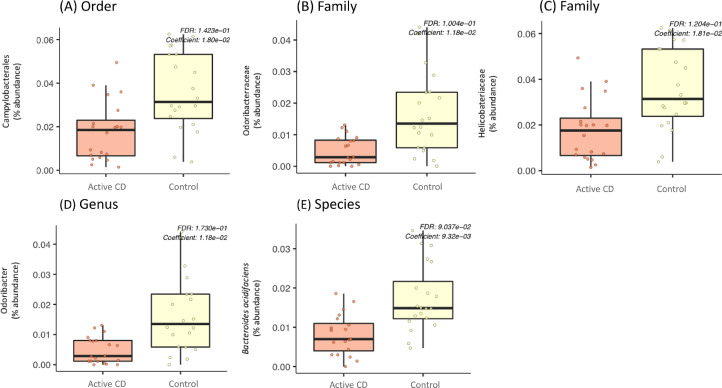
Abundance of taxa that differ between active CD and control subjects. Microbial taxa with significantly different percent abundance between active CD and control subjects at the (A) order, (B + C) family, (D) genus and (E) species levels, (Linear Model, *q* < 0.2).

We evaluated the bacterial communities in the blood samples and compared their composition across subjects with active CD, remission CD, and control groups. Bacteroidetes was found to be the most abundant phylum in all three groups, followed by Firmicutes and Proteobacteria phyla (Fig. 2). Differential abundance analysis identified significant differences between subjects with active CD and controls at multiple taxonomic levels (Fig. 3). In particular, at the order level, the abundance of the Campylobacterales order was decreased in active CD subjects compared to controls (Fig. 3A, *q* = 0.14). At the family level, we identified a decreased abundance of the Odoribacteraceae (Fig. 3B, *q* = 0.10) and Helicobacteraceae (Fig. 3B, *q* = 0.12) families in active CD subjects compared to controls. At the genus level, we found a decreased abundance of the *Odoribacter* genus in active CD subjects when compared to controls (Fig. 3C, *q* = 0.17). Finally, at the species level, there was a decreased abundance of *Bacteroides acidifaciens* species in active CD subjects compared to controls (Fig. 3D, *q* = 0.090). We did not find significant differences between subjects with active and remission CD or remission CD and control subjects.

## 4 Discussion

The purpose of this study was to describe the blood microbiota of children with active CD compared to children with CD in remission and controls. To our knowledge, our findings represent the first characterization of the pediatric blood microbiota. Our study establishes the presence of a pediatric blood microbiota and identifies differences between subjects with active CD and controls.

Across all three groups of samples, the most abundant phylum was Bacteroidetes, followed by Firmicutes, then Proteobacteria. Previous work (Paisse et al., 2016; Whittle et al., 2018; Castillo et al., 2019; Serena et al., 2019) examining the adult blood microbiota has consistently indicated Proteobacteria as the most abundant phylum found in both healthy and diseased (including CD (Serena et al., 2019)) adult blood, with Bacteroidetes, Actinobacteria, and Firmicutes as the next most abundant phyla. These data suggest that the composition of the pediatric blood microbiota differs from that of adults.

Our analysis found significantly higher beta diversity of the blood microbiota in pediatric subjects with active CD compared to control subjects, as measured by Bray-Curtis and Jaccard indices. This indicates that the blood microbiota of active CD patients is significantly different from that of control subjects. Specifically, the Bray-Curtis measure indicates that active CD samples have significantly higher species abundance than healthy controls, while the Jaccard measure indicates that active CD samples have significantly different species from healthy control samples. These results are inconsistent with what was reported in the previous study in adults (Serena et al., 2019), where beta diversity of the blood microbiota in control subjects was higher than in those with active CD, suggesting potential differences between adult and pediatric blood microbiota in health and disease.

We identified differences in the taxonomic composition in the blood microbiota of subjects with active CD compared to control subjects. Specifically, our analysis showed that samples from active CD subjects were characterized by decreased abundance of bacteria belonging to the Campylobacterales order, the Odoribacteraceae and Helicobacteraceae families, the *Odoribacter* genus and species, and the *Bacteroides acidifaciens* species. None of these taxa have been reported in previous blood microbiota studies. We did not find significant differences in the microbiota composition between subjects in remission for CD and those with active CD or with control subjects, consistent with the study of the blood microbiota in adults with CD (Serena et al., 2019).

Given the lack of literature describing the blood microbiota in children and limited data in adults, here we discuss these microbes of interest according to the work available which is predominantly from studies in the gut microbiota. However, the relationship between the blood microbiota and gut microbiota has not yet been elucidated. Therefore, while a connection has been hypothesized (Paisse et al., 2016; Castillo et al., 2019), it is unclear whether the functions of these taxa, previously shown to function in modulating immune response (Faber et al., 2016; Serena et al., 2017; Chang et al., 2014) and associated with gastrointestinal disorders (Zhang et al., 2006; Kaakoush et al., 2010; Parsons et al., 2017; Schwab et al., 2014; Morgan et al., 2012) can be extrapolated from the gut to the blood. For example, we found decreased Campylobacterales in the blood of active CD subjects. Members of this order including *Campylobacter jejuni* and *Campylobacter coli* have been shown to produce a flagellin-like molecule suggested to activate the gut immune response to commensal microbes in the gut (Faber et al., 2016). We also identified decreased abundance of Odoribacteraceae in patients with active CD. This bacterial family produces butyrate (Gomez-Arango et al., 2016) and may serve to decrease inflammation in patients with CD (Serena et al., 2017) through inhibition of histone deacetylase, activation of intestinal macrophages, and downregulation of proinflammatory mediators in the gut (Chang et al., 2014). Decreased abundance of Odoribacteraceae has also been reported in the stool of patients with lupus, an autoimmune condition, compared with controls (Bankole et al., 2017). In addition, consistent with our finding of decreased Campylobacterales, we found decreased Helicobacteraceae, a member of the Campylobacterales order. Bacteria in the Helicobaceraceae family have been shown to be more prevalent in gastric biopsies of children with irritable bowel syndrome and inflammatory bowel disease (Zhang et al., 2006), specifically in intestinal biopsies of children with Crohn's disease (Kaakoush et al., 2010) and increased in gastric biopsies in adults with gastritis (Parsons et al., 2017), when compared to controls. We also found decreased abundance of the genus Odoribacter. These bacteria produce short chain fatty acids, including butyrate (Morgan et al., 2012) and were shown to be decreased in stool and intestinal biopsies of adults with inflammatory bowel disease (Morgan et al., 2012). Finally, we found decreased abundance of *Bacteroides Acidifacien*s. This species has been reported to degrade the intestine's protective mucin layer (Berry et al., 2013) and to be increased in abundance in mice models of colitis (Schwab et al., 2014). While these microbes have been linked with modulating the immune response or linked to autoimmune and inflammatory conditions, these findings are in the setting of the gut microbiota and therefore the meaning of these findings in the blood remains to be determined.

We did not find significant differences in the blood microbiota between active CD and CD in remission subjects or between CD in remission and control subjects, consistent with a study of the blood microbiota in adults with CD (Serena et al., 2019). Previous studies show that patients with CD in remission have gut microbiomes with compositions that in some ways resemble controls, and in other ways resemble the active CD patients (Cenit et al., 2015). Thus, our finding of significant differences between controls and active CD, but none between remission CD subjects’ blood microbiota and the other two groups, is consistent with previous literature.

16S rRNA sequencing is a powerful method that indicated presence of bacterial nucleic acid in healthy and diseased blood (Gosiewski et al., 2017; Paisse et al., 2016; Whittle et al., 2018), but does not indicate presence of living micro-organisms. Bacteria in the blood microbiota are believed to be dormant and are not easy to culture (Castillo et al., 2019). Furthermore, in our study, as in other studies (Castillo et al., 2019), levels of blood bacterial nucleic acid were low. Thus, future work should also focus on bacterial function, developing methods to prove their viability and visualize them in erythrocytes and lymphocytes, where they may reside (Castillo et al., 2019).

Additional limitations of our study include our relatively small sample size of 60 samples and single time point sample collection. Further, although we controlled for several factors such as sex, age, medications, serology, and duodenal histology in determining which patient samples to use, there are a variety of factors such as host genetics, diet, and household size that may affect the microbiome that we did not account for. This allows for the possibility that our findings are not reflective of CD status but instead are reflective of some associated characteristic in these subjects.

Here, we show that 16S bacteria genes are present in blood of children with active CD, CD in remission, and controls. We found that the most abundant phylum in the pediatric blood microbiota across all three subject groups differs from the previously reported most abundant phylum in the adult blood microbiota of subjects with active CD, remission CD, controls, or those with other diseases. We identified significant differences in diversity and abundance of taxa previously shown to be involved with immune response and gut-inflammatory disorders between children with active CD and controls. This could suggest a role for the blood microbiota in the altered immune response that characterizes CD. Our study is an important first step describing the pediatric blood microbiota and in laying a foundation for future work aimed at understanding the potential role it plays in pediatric CD.

## CRediT authorship contribution statement

**Isha Mehrotra:** Investigation, Writing – original draft, Visualization. **Gloria Serena:** Investigation, Writing – review & editing, Supervision. **Murat Cetinbas:** Writing – review & editing, Software, Formal analysis, Data curation. **Victoria Kenyon:** Writing – review & editing, Project administration. **Victoria M. Martin:** Writing – review & editing. **Stephanie G. Harshman:** Writing – review & editing. **Ali R. Zomorrodi:** Writing – review & editing. **Ruslan I. Sadreyev:** Writing – review & editing, Software, Formal analysis, Data curation. **Alessio Fasano:** Writing – review & editing, Funding acquisition, Resources, Methodology. **Maureen M. Leonard:** Investigation, Writing – review & editing, Funding acquisition, Conceptualization, Methodology, Project administration, Supervision.

## Declaration of Competing Interest

The authors declare the following financial interests/personal relationships which may be considered as potential competing interests: Alessio Fasano reports a relationship with Alba Therapeutics that includes: equity or stocks. Alessio Fasano reports a relationship with Innovate Biopharmaceuticals that includes: consulting or advisory. Alessio Fasano reports a relationship with Axial Biotherapeutics that includes: board membership. Alessio Fasano reports a relationship with Viome that includes: board membership. Alessio Fasano reports a relationship with Mead Johnson Nutrition that includes: speaking and lecture fees. Maureen M Leonard reports a relationship with Anokion that includes: consulting or advisory. Maureen M Leonard reports a relationship with 9meters biopharma that includes: board membership. Victoria M Martin reports a relationship with Milk Care Co that includes: board membership.

## Appendix Supplementary materials

Image, application 1

Image, application 2
