# Supplementary material for: Characterization of the blood microbiota in children with Celiac disease
Source: Curr Res Microb Sci. 2021 Aug 30;2:100069. doi: 10.1016/j.crmicr.2021.100069 (PMC8610358; doi:10.1016/j.crmicr.2021.100069)
Supplement: Supplementary file 2 [file mmc2.docx]

## Supplementary Table & Table Legend

**Supplementary Table 1: Study subjects’ characteristics.**

CD = Celiac Disease. M = Males. F = Female. GFD = Gluten Free Diet.

tTG IgA = Tissue TransGlutaminase ImmunoGlobulin A= IgA

*tTG IgA Reference range (0-20).
